# Comparison of Cytotoxicity and Photocatalytic Properties of Iron Vanadate Nanoparticles with Commercial Catalysts: For the Degradation of Microplastics and Bacterial Inactivation Application

**DOI:** 10.1021/acsomega.5c02744

**Published:** 2025-08-11

**Authors:** Guru Karthikeyan Thirunavukkarasu, Paweł Krzyżek, Adityanarayan Mohapatra, Ayeskanta Mohanty, Monika Motlochová, Michal Navrátil, Jaroslav Kupčík, Jan Šubrt, In-Kyu Park, Alicja Seniuk, Ewa Dworniczek

**Affiliations:** † Institute of Inorganic Chemistry of the Czech Academy of Sciences, Husinec-Řež 250 68, Czech Republic; ‡ Department of Microbiology, Faculty of Medicine, 49550 Wroclaw Medical University, Wroclaw 50-368, Poland; § Department of Biomedical Sciences and BioMedical Sciences Graduate Program (BMSGP), 65722 Chonnam National University Medical School, Hwasun 58128, Republic of Korea

## Abstract

Microplastics (MPs) and the development of associated antibiotic-resistant bacteria are of serious concern. Conventional water treatment methodologies do not sufficiently address the issue of MPs and MPs-attached bacteria. The photocatalytic process is a promising technique that utilizes solar light to generate HO^●^ radicals for the degradation of MPs and inactivation of microorganisms. In this work, the iron-vanadate (FeVO_4_, IVAN) nanoparticles prepared by the coprecipitation and a subsequent freeze-drying technique were tested for their cytotoxicity and photocatalytic activity in the degradation of MPs and inactivation of bacteria. Cytotoxicity of the prepared IVAN catalyst showed moderate toxicity levels at a concentration of 12.5 μg/mL. Photocatalytic degradation of catalysts evaluated using attenuated total reflection infrared (ATR-IR) spectroscopy revealed the overall highest increase in the carbonyl index (CI) and peroxyl index (PI) for the IVAN nanoparticles compared with commercial catalysts. The scavenging experiments confirmed that HO^●^ and O_2_^●–^ were the potential main reactive oxygen species produced during the photocatalytic process using IVAN. Furthermore, nuclear magnetic resonance (NMR) spectra proved an oxidative degradation of polystyrene (PS) MPs. Apparently, leaching of Fe and V ions closer to the acceptable toxicity levels was detected by using inductively coupled plasma optical emission spectrometry (ICP-OES). Interestingly, IVAN exhibited inhibition of the *Staphylococcus aureus* USA300 biofilm in both dark and light conditions. Therefore, our investigation of IVAN and commercial photocatalysts could give insights into the preparation of efficient catalysts for treating MPs and bacteria in water.

## Graphical abstract

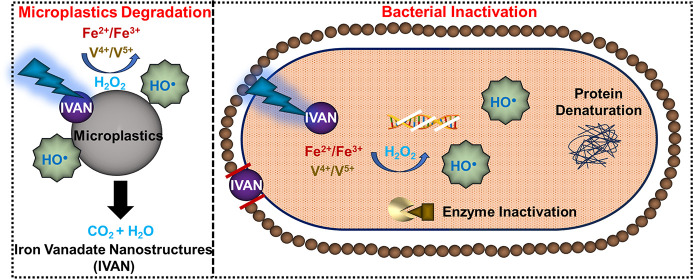

## 1 Introduction

Since plastic product bulk commercialization began in the 1950s, plastic debris has continuously accumulated in all aquatic environments.^1^ With the pace of time, due to the weather and other physical, chemical, and biological processes, this plastic debris might be broken into smaller fragments (microplastics (MPs) and nanoplastics: <5 mm and <1 μm in diameter, respectively).^2–5^ Notably, around 1–2 million metric tons of MPs are annually released into the water bodies.^6^ These MPs are composed of potent chemicals, such as plasticizers, additives, and pigments, all of which pose toxicity to all living organisms.^7^ Furthermore, MPs adsorb organic and inorganic pollutants during their transport cycle, thereby increasing their potency in the ecosystem.^3,8,9^ In particular, the adsorption of antibiotics by MPs is a major concern, since it could lead to the development of antibiotic-resistant genes and the spreading of antibiotic-resistant bacteria in the environment.^10–12^ Currently, available wastewater treatment methodologies are not equipped to treat MPs, which may act as a reservoir for the growth of antibiotic-resistant microorganisms.^13–15^ Furthermore, MPs also promote the transfer of antibiotic-resistance genes in the environment through mobile genetic elements (plasmids, transposons, and others).^13,16^ Therefore, MPs not only cause adverse effects on living organisms but also lead to the development and spread of antimicrobial resistance.^13–15^ Thus, efficient water treatment methodologies are required to degrade MPs and to inactivate bacteria. Generally, coagulation, filtration, adsorption, and advanced oxidation processes are used to remove MPs and bacteria in the environment.^17–24^ For instance, Zhang et al. have developed Cs_3_Bi_2_Br_9_/BiOCl S-scheme junction photocatalysts for the efficient degradation of polystyrene (PS) MPs.^25^ In another study, Li et al. utilized InVO_4_/Bi_5_O_7_I photocatalysts for a simultaneous photocatalytic degradation of organic pollutants (tetracycline and bisphenol A) and inactivation of bacteria (*Staphylococcus aureus* and *Escherichia coli*).^26^ Therefore, the advanced oxidation process, such as the photocatalytic process, can simultaneously degrade MPs into safe compounds and inactivate potentially harmful bacteria.^5,27–33^ Wherein, other treatment methodologies transfer MPs to another medium, but at the same time, cannot inactivate microbes.^27,28,34,35^

Photocatalysis is an extensively applied method of an advanced oxidation process, wherein light of a suitable wavelength is irradiated onto a photocatalyst, triggering the generation of reactive oxygen species (ROS) to degrade organic pollutants as well as to inactivate bacteria.^28,36,37^ Semiconductors, such as TiO_2_, ZnO, MgO, CuO, Fe_2_O_3_, and V_2_O_5_, have been utilized as photocatalysts to treat organic pollutants and bacteria in the environment.^30,37–39^ Furthermore, modified metal oxides, such as TiO_2_/Fe, SnO_2_/Nb, and In_2_O_3_/reduced graphene oxide, were explored for the degradation of MPs.^40–42^ Alternatively, researchers are exploring environmentally friendly metavanadates that exhibit excellent optical properties and high thermal stability for photocatalytic applications.^43–45^ Metal vanadates are typically composed of vanadium in +2/+5 oxidation states, oxygen, and metal elements (Fe, Zn, Mn, or Ni).^43–45^ Among the metal vanadate family, FeVO_4_ nanoparticles (IVAN) are of particular interest due to their narrow band gap (2.0–2.7 eV) and ability to degrade pollutants through photocatalytic and Fenton-based processes.^46,47^ Zhang et al.^48^ reported that depending on the vanadium vacancies-based defects in the band structure, the band gap of the FeVO_4_ structure varies. Furthermore, FeVO_4_ (∼29 ns) exhibits a longer carrier lifetime compared to the TiO_2_ photocatalyst (a few ps).^49,50^ Indeed, hydroxyl radicals (HO^●^) generated through the photocatalytic process and by the photo-Fenton-based reaction of Fe^2+^ and V^5+^ ions with H_2_O_2_ upon incident light irradiation, enable IVAN to degrade organic pollutants.^46,47^

In the current work, IVAN catalysts prepared by the coprecipitation and subsequent freeze-drying techniques were utilized for the photoinduced microplastic degradation and bacterial inactivation. The photoinduced degradation properties of IVAN were compared with commercially available photocatalysts based on TiO_2_-P25, Fe_3_O_4_-Sigma, and V_2_O_5_-Sigma. Furthermore, this work reports for the first time the photoinduced degradation of MPs and bacteria using metal vanadium-based nanostructures.

## 2 Materials and Methods

### 2.1 Chemicals

Iron nitrate hexahydrate (Fe­(NO_3_)_3_·9H_2_O, Sigma-Aldrich), vanadyl sulfate oxide hydrate (VOSO_4_·(H_2_O)_*x*_, Sigma-Aldrich), and ammonia (NH_3_, Penta) were used for the preparation of IVAN. Polystyrene (PS) beads (*M*_w_ ∼192,000, Sigma-Aldrich), dimethylacetamide (DMA, 99%, Sigma-Aldrich), tetrahydrofuran (A.G., Penta), acetone (T.G., Svero Chema), and dialysis bags (Membra-Cel, 44 mm, MWCO-14,000, Roth) were used for the synthesis of PS MPs. The pH of the solution was adjusted by using perchloric acid (HClO_4_, 68%, Lachner) during the photo-Fenton processes. The commercially available catalysts TiO_2_-P25 (Evonik), Fe_3_O_4_ (Sigma-Aldrich), and V_2_O_5_ (Sigma-Aldrich) were used for the photoinduced degradation of MPs.

### 2.2 Characterization Techniques

Crystalline phases of TiO_2_-P25, Fe_3_O_4_-Sigma, V_2_O_5_-Sigma, and IVAN were analyzed by X-ray diffraction (XRD, Co radiation; Malvern PANalytical Empyrean III and Cu radiation; PANalytical X’pert Pro Multi-Purpose Diffractometer). The shape, morphology, and chemical composition of the samples were analyzed using a high-resolution scanning electron microscope (HRSEM, FEI Nova NanoSEM 450 microscope). HRSEM was equipped with an Everhart–Thornley secondary electron detector, a through-lens detector, and an energy-dispersive X-ray (EDX) analysis detector. Only IVAN was imaged using a high-resolution transmission electron microscope (HR-TEM, Talos F200X, Thermo Scientific) at an accelerating voltage of 200 kV equipped with a field-emission gun. The HR-TEM has combined scanning TEM (STEM) and TEM imaging fitted with 4 in.-column SDD Super-X windowless detectors for EDX chemical analysis and elemental mapping. A diluted suspension of IVAN was gently dropped onto a standard TEM grid covered with carbon foil and then dried at ambient temperature before HR-TEM imaging. The photoinduced degradation of MPs was evaluated using spectra recorded from an attenuated total reflection infrared (ATR-IR) spectrometer (Nicolet Nexus 670) on a diamond crystal in the region from 400 to 4000 cm^–1^. Proton (^1^H) nuclear magnetic resonance spectra were measured by using a nuclear magnetic resonance (NMR, JEOL 600 MHz) spectrometer. The samples (∼20 mg/mL) after the photocatalytic treatment process were suspended in d-chloroform (99.8%, Acros Organics), followed by sonication for 1 min to extract PS MPs. Then, the samples were centrifuged to remove the photocatalysts, and subsequently, the supernatant solution containing dissolved PS MPs was measured in an NMR spectrometer.

### 2.3 Synthesis of IVAN

IVAN catalysts were prepared by a simple precipitation and a freeze-drying technique reported in our previously published works on titanium-based aerogel powders.^51–54^ Accordingly, the equimolar concentrations of iron­(III) nitrate hexahydrate (204 mM) and vanadyl sulfate oxide hydrate (204 mM) were dissolved in 150 mL of distilled water at room temperature until a bluish-black solution was obtained. Then, the solution was kept in the freezer until a thin layer of ice formed, and subsequently, ammonia solution was added until the pH reached 8. Finally, the precipitate was stirred for 1 h, followed by three centrifugation cycles to remove the sulfate ions and excess ammonia. The obtained pellets were dried at room temperature and then annealed at 600 °C (2 °C/min) for 1 h to form the IVAN catalysts. The prepared IVAN catalyst’s shape, morphology, crystallinity, and surface properties were thoroughly characterized.

### 2.4 Synthesis of the PS MPs

PS MPs prepared by the dialysis technique were used as a model pollutant.^55,56^ Typically, approximately 85 mg of PS beads (three beads) were dissolved in 30 mL of organic solvent mixture (10 mL of DMA, 10 mL of tetrahydrofuran (THF), and 10 mL of acetone) and subsequently poured into a dialysis bag. Later, the dialysis bag was immersed and stirred for 24 h in 1.5 L of distilled water (replaced five times) to form an aqueous suspension of PS MPs. Finally, the aqueous PS MP suspension was removed from the dialysis bag and resuspended in 300 mL of distilled water. The final concentration of the PS MPs in the 300 mL suspension would be approximately 0.1 mg/mL. Freshly prepared PS MPs were used for all of the photoinduced degradation experiments.

### 2.5 In Vitro Cytotoxicity Assay

To investigate the in vitro cytotoxicity of the photocatalysts, a WST assay (Ab Frontier Cellvia) was conducted. In brief, 1 × 10^4^ fibroblast cells (L929) and colon cancer cells (CT26) were cultured in a 96-well plate with Dulbecco’s modified Eagle medium (DMEM) and incubated overnight at 37 °C in a 5% CO_2_ atmosphere. After incubation, the medium was removed and the cells were treated with various concentrations of the samples in a fresh DMEM solution, followed by 24 h of culture. Cellular viability was subsequently measured via the WST assay using a microplate reader.

### 2.6 Photoinduced Degradation Experiments

The photoinduced degradation (photocatalysis and photo-Fenton process) experiments were performed by adding 60 mg of photocatalyst to 300 mL of PS microplastic suspensions (0.1 mg/mL). The photocatalyst–PS MP mixture was irradiated for 480 min with constant stirring and air bubbling under 365 nm (λ_max_) UVA light (8 W, Sylvania) at a flux density of 6.24 mW/cm^2^ measured by UVa probe #28949; ILT 1400-A Photometer. The samples were collected before treatment (0 min) and after photoinduced degradation (480 min), then filtered and dried at room temperature for ATR-IR analysis.

### 2.7 Evaluation of PS MP Degradation

ATR-IR spectroscopy was used to calculate the carbonyl index (CI) and peroxyl index (PI), respectively.^27,28,57^ The CI and PI values indicate the oxidative degradation of MPs, i.e., cleavage of the carbonyl (1740 cm^–1^) and peroxyl (1150 cm^–1^) chains during the photoinduced degradation process. Furthermore, the reference peak at 2850 cm^–1^ was used as an internal standard for PS MPs, corresponding to the C–H stretching vibrations of the CH_2_ groups. CI and PI were calculated from the following equations

$$CI=\frac{height\;of\;the\;peak\;at\;\mathrm{1740}\;cm^{-1}}{height\;of\;the\;reference\;peak\;at\;2850\;cm^{-1}}$$

$$\mathrm{PI}=\frac{\mathrm{height}\;\mathrm{of}\;\mathrm{the}\;\mathrm{peak}\;\mathrm{at}\;1150\;cm^{-1}}{\mathrm{height}\;\mathrm{of}\;\mathrm{the}\;\mathrm{reference}\;\mathrm{peak}\;\mathrm{at}\;2850\;cm^{-1}}$$

Importantly, the spectra were normalized at 2850 cm^–1^ for an equivalent comparison of all of the measured spectra. The height of the peaks was calculated by using the Gaussian fitting.

### 2.8 Antimicrobial Testing

The overnight (18 h) culture of *S. aureus* ATCC BAA-1556 (USA300) on Tryptic Soy Agar (TSA, Biomaxima, Poland) was suspended in 5 mL of NaCl (0.85%) to establish an optical density (OD) equivalent to 0.5 McFarland standard ((1–2) × 10^8^ CFU/mL).

To obtain TiO_2_-P25, V_2_O_5_-Sigma, Fe_3_O_4_-Sigma, and FeVO_4_ (IVAN) final concentrations from 8 to 1024 μg/mL, serial dilutions of 2× concentrated powders were prepared in 1 mL of a 0.85% NaCl solution. Suspensions prepared in this manner were then mixed with 1 mL of bacterial solutions diluted 100-fold in a 0.85% NaCl solution. An untreated culture of *S. aureus* was used as the negative control. Prepared samples (mixtures of the bacteria and tested compounds) were preincubated at room temperature in the dark for 0.5 h. Then, half of the samples were exposed to the light of a xenon lamp (50 mW/cm^2^, 35 W, Optel, Poland) in a cooling system for 60 min. The second half of the samples were incubated for 60 min in the dark. Then, following 10-fold dilutions up to 10^6^ in a 0.85% NaCl solution, 50 μL of each sample was plated in triplicate in Petri dishes containing the TSA medium. The plates were incubated overnight at 37 °C. The colony numbers were counted (CFU/mL). The final results were expressed as the percentage of bacterial cells that survived inactivation with a given photocatalyst. The experiment was repeated three times.

### 2.9 Real-Time Assessment of the Antibiofilm Activity

The real-time impact of the tested nanoparticles on the preformed *S. aureus* biofilm was determined using the xCELLigence RTCA DP (Agilent, San Diego, CA) as previously described.^58,59^ The xCELLigence can measure variations in the impedance signal in the dedicated 16-well E-plates (Agilent) with built-in gold microelectrodes. All experiments were recorded by RTCA Software PRO version 2.6.1 (Agilent), and the data were presented as baseline cell index (BCI) values, which detected the amount of biofilm attached.

First, to develop the *S. aureus* biofilm in the 16-well E-plates, each well was filled with 200 μL of a bacterial suspension in MHB with a density of 0.5 McFarland units (1–2) × 10^8^ CFU/mL. For the negative control, 200 μL of pure MHB was added to each well. The xCELLigence system, along with the accordingly prepared E-plates, was placed in a MaxQ 6000 incubator (ThermoFisher, Waltham, MA) and incubated for 24 h at 37 °C to allow *S. aureus* to form a biofilm on the plates. After this period, the wells of the E-plates were emptied, rinsed once with 200 μL of a 0.85% NaCl solution, and then refilled with 200 μL of MHB with 1024 μg/mL of one of the tested nanoparticles. Wells containing a pure MHB served as a negative control (during the initial validation, it was determined that the tested nanoparticles did not affect the impedance readings). The E-plates were incubated for 0.5 h in the darkroom at room temperature to allow the nanoparticles to penetrate the biofilm structure. Then, the E-plates in the photocatalytic model were exposed for 1 h to xenon light at 50 mW/cm^2^, 35 W, and a distance of 10 cm (Optel), while the E-plates in the nonphotocatalytic model were kept in the same environmental conditions but without xenon light exposure. After this stage, the E-plates were returned to the xCELLigence system, and a 24-h incubation at 37 °C was initiated. During this stage, changes in the impedance were automatically measured at 15 min intervals. All experiments were performed in three biological repetitions.

The obtained results were presented as a graph showing BCI values of the nanoparticle-exposed biofilm over time. All impedance readings were normalized to the results of the negative control. Negative BCI values (≤−0.1) indicated degradation of the preformed biofilm, BCI values close to 0 ± 0.1 suggested inhibitory activity on biofilm development without the ability to disrupt its structure, while positive BCI values (≥0.1) showcased further growth of the biofilm biomass.

## 3 Results and Discussion

HR-TEM was used to observe IVAN’s morphology using a high-resolution transmission microscope (Figure 1a). IVAN was composed of a nanoparticle-like morphology with a particle size ranging from 40 to 100 nm. Furthermore, IVAN and commercially purchased photocatalysts were imaged using a high-resolution scanning electron microscope (HRSEM, Figure S1), revealing a nanoparticle-like morphology for IVAN, TiO_2_-P25, and Fe_3_O_4_-Sigma, and a leaflet-like morphology for V_2_O_5_-Sigma. Figure 1b shows that the XRD diffraction results IVAN was majorly composed of the FeVO_4_ crystallite structure (reference code: 00-038-1372) with a minor presence of hematite Fe_2_O_3_ (reference code: 00-024-0072). TiO_2_-P25 comprised 84% anatase (01-084-1286) and 16% rutile (01-078-1510) phase, respectively. V_2_O_5_-Sigma had an orthorhombic V_2_O_5_ phase structure (reference code: 01-077-2418). Fe_3_O_4_-Sigma consisted of a majority magnetite phase (01-075-1609) with a small presence of the maghemite (Fe_2_O_3_) phase (00-015-0615). The photocatalysts had varying crystallite sizes, which were calculated by using the Scherrer equation (Table S1). The crystallite sizes were 24.3, 32.5, 75.4, and 45.3 nm for TiO_2_-P25, Fe_3_O_4_-Sigma, V_2_O_5_-Sigma, and IVAN, respectively. Apparently, the crystallite size calculated from the XRD diffractogram estimates only the average size of the nanomaterials; however, the actual shape or size of the nanomaterials may vary.^60–62^ Further, the surface area of the photocatalysts was measured using the Brunauer–Emmett–Teller (BET) N_2_ adsorption/desorption isotherm method (Figure S2, Table S1). The surface areas of the photocatalysts were 47.2, 32.9, 3.5, and 16.2 m^2^/g, respectively, for TiO_2_-P25, Fe_3_O_4_-Sigma, V_2_O_5_-Sigma, and IVAN. Among the tested photocatalysts, TiO_2_-P25 had the lowest crystallite size and higher surface area, whereas V_2_O_5_-Sigma had the highest crystallite size and lowest surface area among the tested catalysts. Whereas, Fe_3_O_4_-Sigma and IVAN had the median range of crystallite size and surface area. However, it is essential to have an optimum range of particle size, crystallite size, and surface area along with a reduced e^–^/h^+^ recombination process to achieve efficient photocatalytic activity.^63^

**Figure 1:**
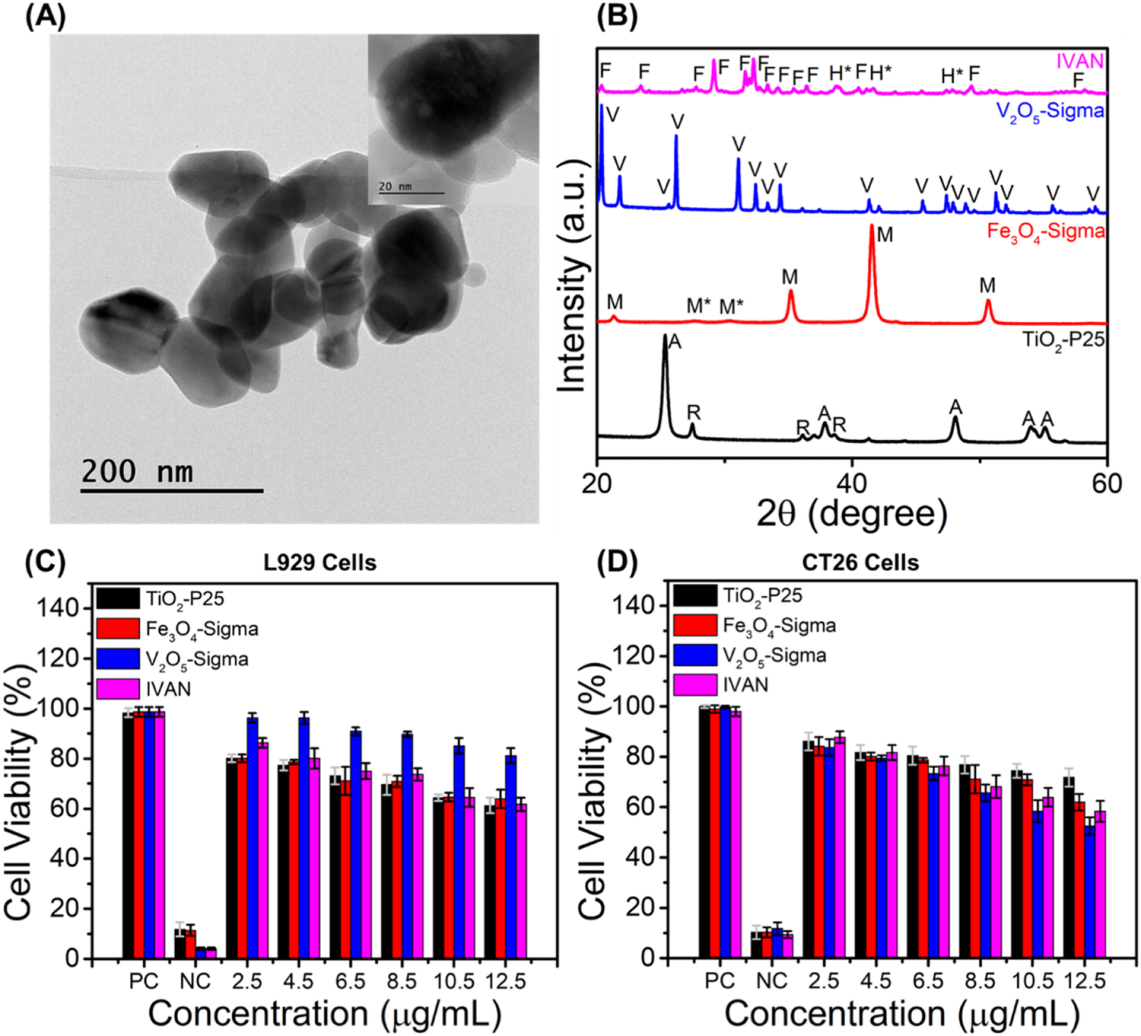
(A) Representative HR-TEM image of IVAN. The inset represents a high-magnification image of the individual nanoparticle. (B) XRD patterns of TiO_2_-P25, Fe_3_O_4_-Sigma, V_2_O_5_-Sigma, and IVAN, respectively. The labels F represent FeVO_4_, H* represents hematite phase Fe_2_O_3_, V represents V_2_O_5_, M represents Fe_3_O_4_, M* represents maghemite phase Fe_2_O_3_, A represents anatase TiO_2_, and R represents rutile TiO_2_, respectively. (C, D) Cytotoxicity tests of TiO_2_-P25, Fe_3_O_4_-Sigma, V_2_O_5_-Sigma, and IVAN using fibroblast cells (L929) and colon cancer cells (CT26).

Cytotoxic effects of the photocatalysts of TiO_2_-P25, Fe_3_O_4_-Sigma, V_2_O_5_-Sigma, and IVAN were investigated by a WST assay using fibroblast (L929) and colon cancer (CT26) model cell lines (Figure 1c,d). At the lowest tested concentration of 2.5 μg/mL, all of the photocatalysts reduced the cell viability of both cell lines to approximately 80%. However, increasing the catalyst concentration led to decreased cell viability. At the highest tested concentration of 12.5 μg/mL, this parameter dropped to ∼60% for both cell lines. Interestingly, L929 cells maintained an 80% survival rate after exposure to the highest tested concentration (12.5 μg/mL) of V_2_O_5_-Sigma, whereas under the same conditions, the viability of CT26 cells decreased by half. This contrasting difference in the cytotoxicity can be attributed to the variable physical–chemical properties of the nanomaterials (particle size, surface charge, and hydrophobicity), which in turn affect their cellular uptake and cell viability.^64,65^ Ivanković et al.^66^ reported that the V_2_O_5_ nanoparticles effectively reduced the cell viability of L929 and FsaR cells, whereas resistance to the nanoparticle treatment was observed for squamous carcinoma SCCVII cells. According to the ISO 10993-5:2009 cell toxicity standards, the survival rate of viable cells above 80% was considered noncytotoxic, while the values of 60–80, 40–60% and less than 40% were considered as moderately weakly cytotoxic, moderately cytotoxic, and cytotoxic, respectively.^67–69^

Photoinduced degradation of MPs was evaluated using ATR-IR spectroscopy, which analyzed changes in the carbonyl and peroxyl indices (CI and PI) compared to nontreated samples (Figures 2A, S3, and Table S2). Briefly, CI and PI values were evaluated by calculating the ratio of the peak heights at 1740 cm^–1^ (carbonyl group) and 1150 cm^–1^ (peroxyl group) to the peak height at 2850 cm^–1^ (internal reference peak of −CH_2_– group). Typically, the oxidation of PS MPs leads to chain scission at the carbonyl, peroxyl, and hydroxyl groups as well as polymer cross-linking.^70,71^ Photoinduced degradation of PS MPs occurs due to the formation of HO^●^ (hydroxyl radical), ROO^●^ (peroxyl radical), and POO^●^ (polystyryl radicals).^70,72^ HO^●^ radicals are produced during the photocatalytic and photo-Fenton-based process, whereas POO^●^ radicals and ROO^●^ radicals are produced during the degradation of PS.^39,51,72–74^ PS MPs break down in various ways, creating different intermediates and several polymer moieties.^72^

**Figure 2:**
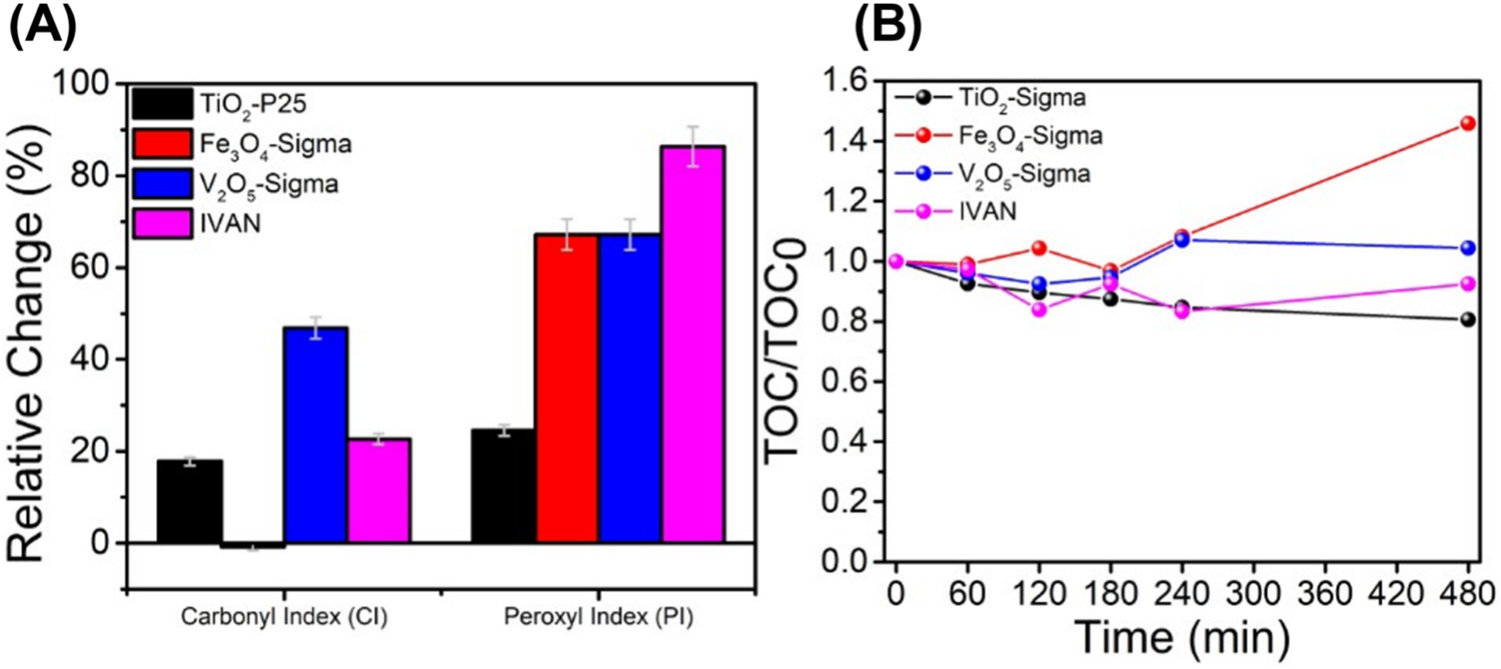
(A) Relative change (%) in the CI and PI values after the photocatalytic process was calculated from the ATR-IR spectra for TiO_2_-P25, Fe_3_O_4_-Sigma, V_2_O_5_-Sigma, and IVAN, respectively, after the photocatalytic process. (B) Total organic carbon (TOC) content was monitored throughout the photocatalytic degradation process.

Accordingly, the photocatalytic degradation of PS MPs using TiO_2_-P25, Fe_3_O_4_-Sigma, V_2_O_5_-Sigma, and IVAN showed varied changes in the CI and PI values. TiO_2_-P25 resulted in approximately 18% change in the CI and approximately 25% change in PI values, respectively. Then, Fe_3_O_4_-Sigma showed a negligible change in the CI value, whereas V_2_O_5_-Sigma showed the highest change in the CI value to approximately 46%. Interestingly, Fe_3_O_4_-Sigma and V_2_O_5_-Sigma had a similar PI value. IVAN had a higher overall change in indexes with approximately 22 and 86% change in CI and PI values, respectively. Therefore, it can be observed that chain scissions occur in different groups (carboxyl and peroxyl) depending on the photocatalyst. Further, total organic carbon (TOC) content analysis revealed that the approximately 20 and 8% mineralization were observed for TiO_2_-P25 and IVAN, respectively (Figure 2B). However, Fe_3_O_4_-Sigma and V_2_O_5_-Sigma did not mineralize the PS MPs, as deduced by their inability to convert PS MPs to CO_2_ and H_2_O. The samples before and after the photocatalytic process were imaged by using HRSEM with EDX mapping (Figure S4). The scavenging experiments were conducted to determine the reactive oxygen species responsible for the degradation of PS MPs using IVAN (Figure S5). Therefore, photocatalytic experiments were performed using isopropyl alcohol (IPA, HO^•^) and *p*-benzoquinone (BQ, O_2_^•–^, and ^1^O_2_) as scavengers. There was a clear reduction in CI and PI values with the use of the scavengers. Hence, we could assume that HO^•^ and O_2_^•–^ radicals play a role in the degradation of the PS MPs. Especially, O_2_^•–^ reacts with H^+^ to form H_2_O_2_, which was either decomposed or utilized in the Fenton process (with Fe^2+^/Fe^3+^ or V^4+^/V^5+^) to form HO^•^ (Figure S6). The produced HO^•^ radicals scission the polymer chains to form esters, phenols, and organic acids as byproducts and would eventually be converted to CO_2_ and H_2_O when IVAN was irradiated for a longer time.

Additionally, nuclear magnetic resonance (NMR) spectra revealed the oxidative degradation of PS MPs (Figure S7). The peaks between 7.20 and 6.45 ppm belong to the styrene moieties, and those between 1.9 and 0.8 ppm belong to the aliphatic protons.^75^ At 5.00 and 2.3 ppm, protons belonging to the aromatic alcohol were present due to the oxidation of PS MPs that occurred during the synthesis of PS MPs.^75^ Clear changes in the NMR spectra were observed with respect to the PS MPs and after the photoinduced treatment process using TiO_2_-P25, V_2_O_5_-Sigma, and IVAN. However, NMR could not be measured for the samples with Fe_3_O_4_-Sigma due to its magnetic nature. Nevertheless, indications of the oxidative degradation process were observed through changes in the peaks related to styrene moieties, aromatic alcohols, and aliphatic protons in the NMR spectra of the other tested photocatalysts.

Furthermore, the leaching of metal ions into the solution after the photoinduced degradation process was monitored using inductively coupled plasma optical emission spectrometry (ICP-OES) and tabulated in Table 1. Ti ions were leached in trace quantities from all of the photocatalysts. Especially, the leaching of Ti ions in non-TiO_2_-based photocatalysts could be associated with the impurity present in the precursors. However, the approximately 86 μg/mL leaching of V ions from V_2_O_5_-Sigma was significantly higher than the cytotoxicity safety limits (Figure 1c,d). Therefore, even though V_2_O_5_-Sigma could potentially degrade the MPs, the toxicity of V ions could limit its use. Fe and V ions leaching for IVAN after the photocatalytic degradation process were close to the moderate cytotoxicity limits. Moreover, we assume that the Fe and V ions were leached in the form of oxides of Fe/V as observed from the XRD pattern after the three cycles of the photocatalytic process (Figure S8). Apparent changes in the crystal structure of IVAN were observed after the photocatalytic process. Although the IVAN degraded, it still degraded into another form of the photocatalyst. We also tested the cyclic stability of IVAN after three photocatalytic cycles (data not included). However, we observed that lower CI and varying PI values occurred after each photocatalytic cycle. Since the cyclic stability experiment requires the separation of degraded PS MPs and photocatalysts after each degradation cycle via extraction with chloroform. The extraction step hindered the photocatalytic abilities of IVAN. Nevertheless, IVAN could potentially be used as a photocatalyst for the degradation of MPs and the inactivation of bacteria.

**Table 1:** Investigation of Leaching of Metal Ions (Fe, Ti, and V) after the Photoinduced Degradation of MPs through the ICP-OES Technique

sample name	Fe (238.204 nm) (μg/mL)	Ti (336.122 nm) (μg/mL)	V (268.796 nm) (μg/mL)
TiO_2_-P25	0.000	1.616	0.000
Fe_3_O_4_-Sigma	0.409	0.012	0.000
V_2_O_5_-Sigma	0.029	0.018	85.727
IVAN	14.059	0.028	11.965

To assess the antimicrobial properties of the IVAN, V_2_O_5_-Sigma, Fe_3_O_4_-Sigma, and TiO_2_-P25 photocatalysts, they were first tested against planktonic forms of *S. aureus* USA300, exposed to xenon lamp irradiation (Figure S9A) and under dark conditions (Figure S9B). The examined photocatalysts exhibited antimicrobial activity under both tested conditions. After 60 min of irradiation, there was a slight decrease (∼20%) in the survival of planktonic staphylococci exposed to higher concentrations of Fe_3_O_4_-Sigma, V_2_O_5_-Sigma, and IVAN photocatalysts. The survival of *S. aureus* USA300 was the lowest after photocatalytic inactivation with TiO_2_-P25. In this case, the decrease in bacterial survival reached 100% at a concentration of 512 μg/mL.

In the following research stage, the influence of the photocatalyst (1024 μg/mL) on the biomass of preformed *S. aureus* USA300 biofilms was investigated using real-time impedance measurements (Figure 3). Both research models, with and without xenon light irradiation, yielded convergent results. In the photocatalytic model, biofilm samples in the control setting (*S. aureus* biofilm) and those exposed to Fe_3_O_4_-Sigma exhibited baseline cell indexes (BCI) of 0.256 and 0.227, respectively. Contrary to this, biofilm development was inhibited by the rest of the tested photocatalysts (BCI in the range of 0.05–0.073). In the nonphotocatalytic model (dark conditions), control biofilm samples and those treated with Fe_3_O_4_-Sigma grew to a BCI of 0.212 and 0.12, respectively. In contrast, for IVAN and TiO_2_-P25, inhibitory activities against biofilm development were detected (BCI values of −0.036 and −0.064, respectively). Additionally, V_2_O_5_-Sigma was observed to have the ability to disrupt the preformed biofilm of *S. aureus* USA300, as indicated by a BCI value of −0.151. Notably, in dark conditions, the degree of antibiofilm activity of IVAN, TiO_2_-P25, and V_2_O_5_-Sigma was more noticeable (after 24 h of incubation, the impedance signal was below the detection threshold) than in the light conditions using photocatalysts, where a slow but steady progression of biofilm development began to occur between 16 and 18 h of culture.

**Figure 3:**
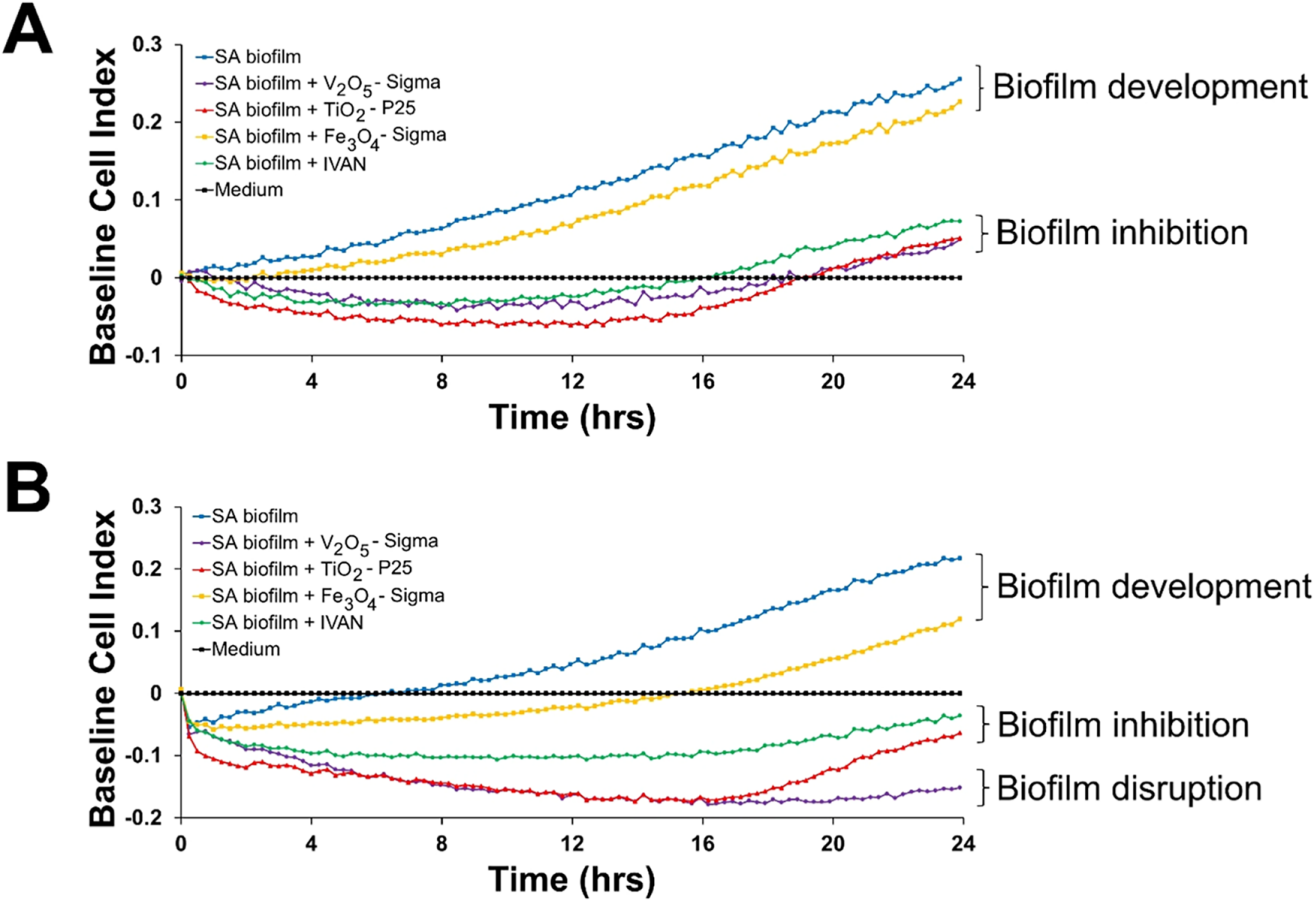
Antibiofilm activity of the tested nanoparticles (V_2_O_5_-Sigma, TiO_2_-P25, Fe_3_O_4_-Sigma, and IVAN) against a 24-h, preformed biofilm of *S. aureus* USA300. Tests were performed after the xenon lamp irradiation (A) and in dark conditions (B). Changes in biofilm production were measured by the xCELLigence system. Negative BCI values (≤−0.1) indicate degradation of the preformed biofilm. BCI values close to 0 ± 0.1 suggest inhibitory activity on biofilm development without the ability to disrupt its structure, while positive BCI values (≥0.1) showcase further growth of the biofilm biomass. All results represent the average of three biological replicates (*n* = 3).

The results of microbiological tests conducted for *S. aureus* USA300 indicate its moderate susceptibility to inactivation under the conditions we used. Staphylococci are thick-walled bacteria with features that enable them to survive in the external environment, including the harsh hospital environment.^76,77^ Additionally, *S. aureus* USA300 exhibits an MRSA phenotype with broad resistance to β-lactam antibiotics.^78^ It cannot be excluded that the moderate antibacterial effect of IVAN and V_2_O_5_-Sigma, Fe_3_O_4_-Sigma, and TiO_2_-P25 has been associated with the specific nature of the investigated bacteria, as demonstrated in our pilot examination. In the future, extended studies with a larger group of microorganisms would be necessary to elucidate the antimicrobial mechanisms.

In summary, the commercial photocatalysts (TiO_2_-P25, Fe_3_O_4_-Sigma, and V_2_O_5_-Sigma) and IVAN exhibited varying abilities to degrade MPs and prevent bacterial growth. In this regard, V_2_O_5_-Sigma exhibited higher leaching of V^+^ ions, which could cause toxicity to living organisms and therefore cannot be considered for real-time applications. Fe_3_O_4_-Sigma did not demonstrate sufficient photocatalytic effects. Meanwhile, TiO_2_-P25 and IVAN exhibited acceptable toxicity levels, which considerably induced the oxidative degradation of MPs and prevented the development of the *S. aureus* USA300 biofilm, even without the application of light. Therefore, IVAN could be a promising alternative catalyst for treating water containing organic pollutants and harmful microorganisms.

## 4 Conclusions

IVAN nanoparticles were successfully synthesized and tested for their cytotoxicity and photocatalytic properties. IVAN had a moderate level of toxicity with a cell viability of ∼65% at the highest concentration of 12.5 μg/mL. Photocatalytic degradation of PS MPs using IVAN resulted in the highest change in CI and PI values of approximately 22 and 86%, respectively. Subsequently, the oxidative degradation of PS MPs was confirmed by NMR spectra, which showed clear changes before and after the photocatalytic process, particularly at 2.3, 5.0, and 1.9–0.8 ppm. Furthermore, IVAN inhibited the further development of *S. aureus* USA300 preformed biofilm in both photocatalytic (light conditions) and nonphotocatalytic (dark conditions). Therefore, our investigation using IVAN and commercial catalysts could pave the way for exploring less toxic photocatalysts for the degradation of MPs and bacterial inactivation applications.

## Supplementary Material



## Notes

Crystallite size, surface area, and HRSEM images of the photocatalysts, ATR-IR spectra, calculated CI and PI values after the photocatalytic process, HRSEM with EDX mapping after the photocatalytic process, scavenging experiments, production of ROS and PS MP degradation mechanism, XRD after three photocatalytic cycles, NMR spectra after the photocatalytic process, and antibacterial activity in planktonic forms of *S. aureus* bacteria (PDF)
