# Supplementary material for: Comparison of Cytotoxicity and Photocatalytic Properties of Iron Vanadate Nanoparticles with Commercial Catalysts: For the Degradation of Microplastics and Bacterial Inactivation Application
Source: ACS Omega. 2025 Aug 11;10(33):37284–93. doi: 10.1021/acsomega.5c02744 (PMC12392196; doi:10.1021/acsomega.5c02744)
Supplement: Supplementary file 1 [file ao5c02744_si_001.pdf]

Comparison of cytotoxicity and photocatalytic properties of iron vanadate nanoparticles with commercial catalysts: For the degradation of microplastics and bacterial inactivation application

Guru Karthikeyan Thirunavukkarasu[a*], *Paweł Krzyżek*[b], *Adityanarayan Mohapatra*[c], *Ayeskanta Mohanty*[c], *Monika Motlochová*[a], *Michal Navrátil*[a], *Jaroslav Kupčík*[a], *Jan Šubrt*[a], *In-Kyu Park*[c], *Alicja Seniuk*[b], *Ewa Dworniczek*[b*]

[a] Institute of Inorganic Chemistry of the Czech Academy of Sciences, Husinec-Řež 250 68, Czech Republic

[b] Department of Microbiology, Faculty of Medicine, Wroclaw Medical University, Wroclaw 50-368, Poland

[c] Department of Biomedical Sciences and BioMedical Sciences Graduate Program (BMSGP), Chonnam National University Medical School, Hwasun 58128, Republic of Korea

*Corresponding author: thirunavukkarasu@iic.cas.cz (G.K. Thirunavukkarasu) and ewa.dworniczek@umw.edu.pl (E. Dworniczek)

Table S1: Crystallite sizes of the photocatalysts were calculated from the XRD diffractograms using the Scherrer calculator from the X'pert HighScore software. Surface Area, Pore Volume, and Pore Diameter were measured using the Surface Area Analyzer (Quantachrome Nova 4200e).

**Sample**	**Crystallite Size (nm)**	**Surface Area (m^2/g)**
TiO_2-P25	24.3	47.2
Fe_3O_4-Sigma	32.5	32.9
V_2O_5-Sigma	75.4	3.5
IVAN	45.3	16.2

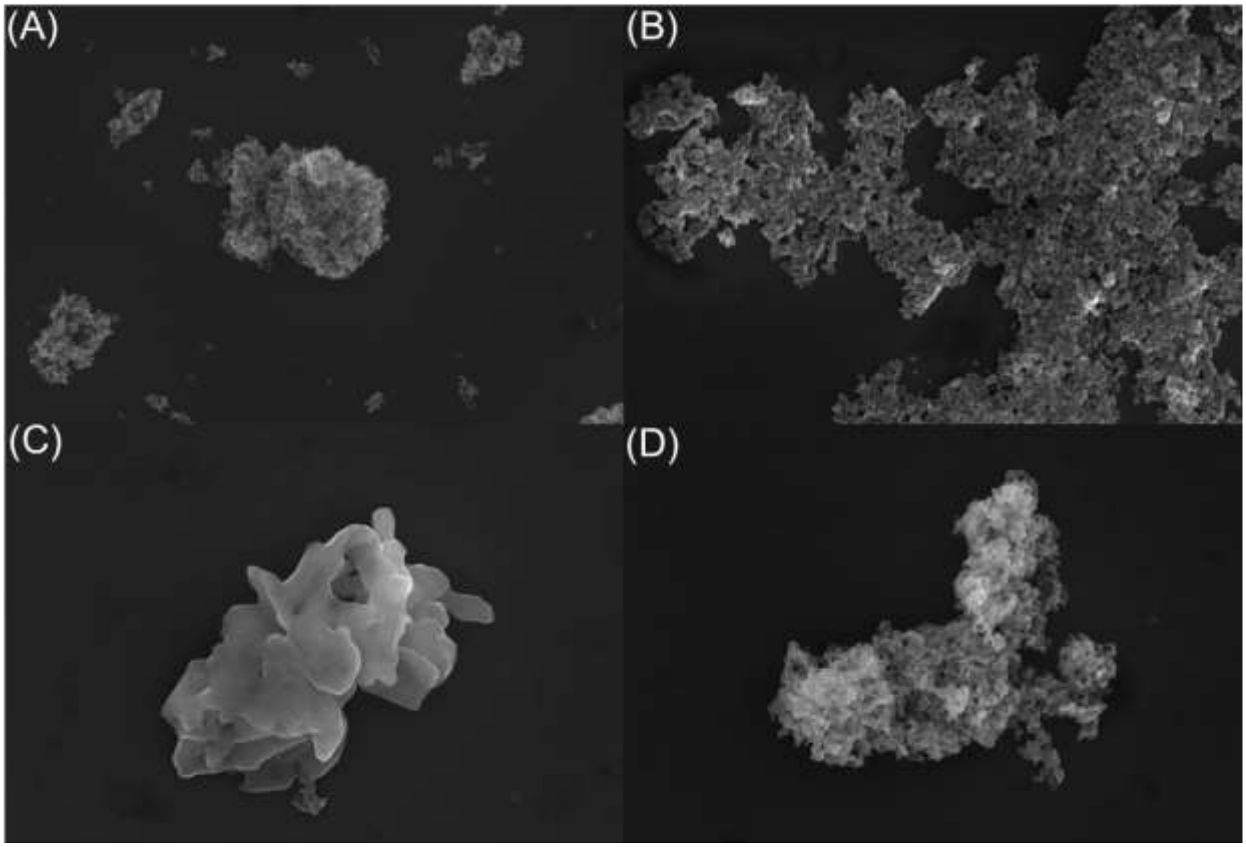

Figure S1: Representative HRSEM images of commercially available (A) TiO_2-P25, (B) Fe_3O_4-Sigma, (C) V_2O_5-Sigma, and (D) IVAN.

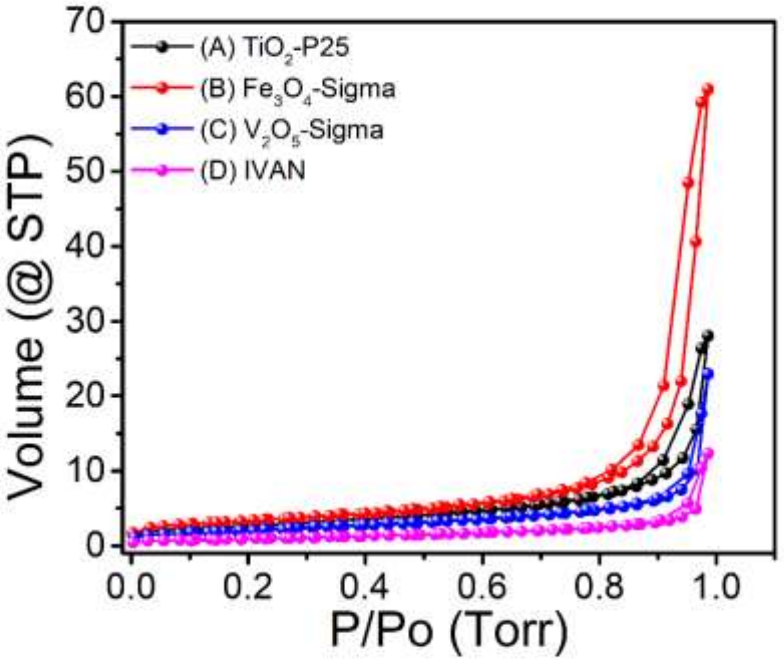

Figure S2: Brunauer–Emmett–Teller (BET) adsorption–desorption isotherm of (A) TiO_2-P25, (B) Fe_3O_4-Sigma, (C) V_2O_5-Sigma, and (D) IVAN.

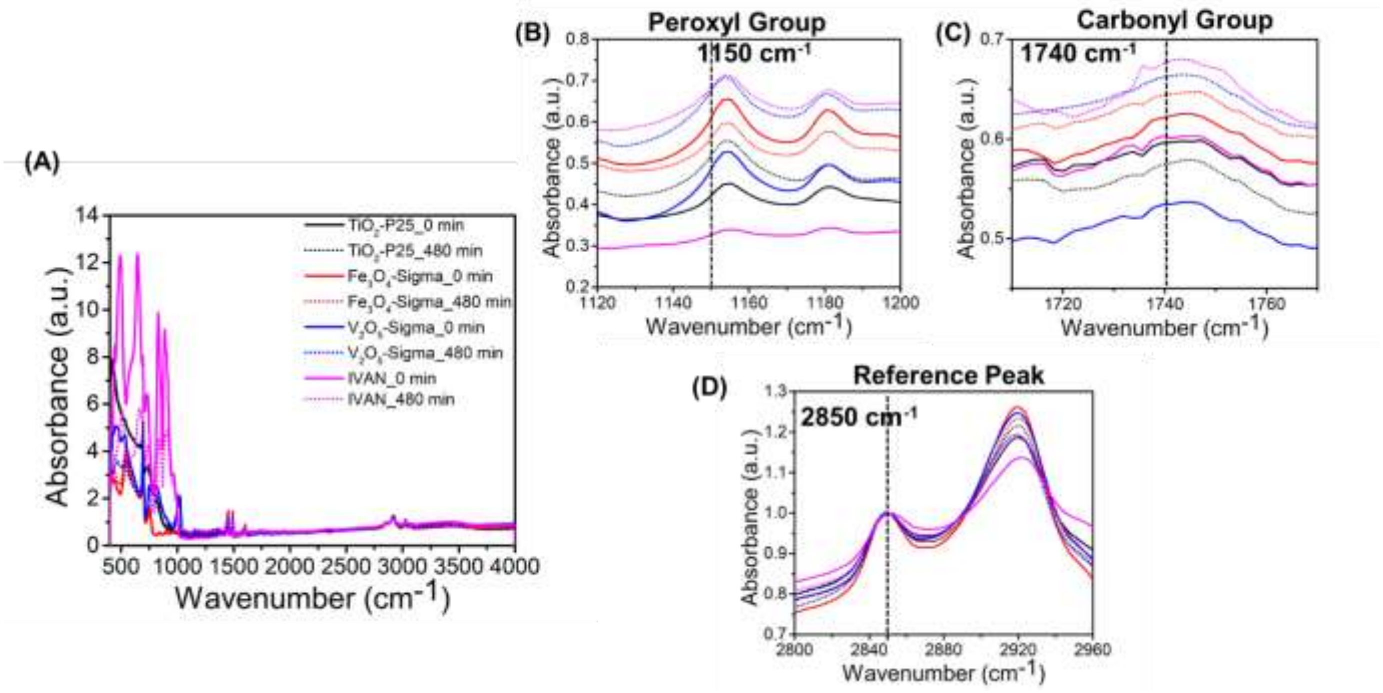

Figure S3: (A) Shows the changes in ATR-IR spectra before (0 min) and after (480 min) the photocatalytic process for the photocatalysts TiO_2-P25, Fe_3O_4-Sigma, V_2O_5-Sigma, and IVAN.

On the right side, zoomed-in spectra of the peroxyl group at 1150 cm^{-1}(B), carbonyl group at 1740 cm^{-1} (C), and reference group at 2850 cm^{-1} (D), respectively.

Table S2: Calculated CI and PI from the ATR-IR spectra for all the tested photocatalysts are tabulated. The relative change in CI and PI are calculated from these values. Error is ± 5%.

Sample	Carbonyl Index (CI)	Peroxyl Index (PI)
TiO_2-P25 (Before treatment)	0.232387139	0.317891141
TiO_2-P25_480 min	0.273778549	0.395971861
Fe_3O_4-Sigma (Before treatment)	0.227374746	0.228414336
Fe_3O_4-Sigma_480 min	0.225511811	0.381889764
V_2O_5-Sigma (Before treatment)	0.161972188	0.353127093
V_2O_5-Sigma_480 min	0.237860892	0.58716098
IVAN (Before treatment)	0.260673425	0.260015322
IVAN_480 min	0.319788397	0.484594815

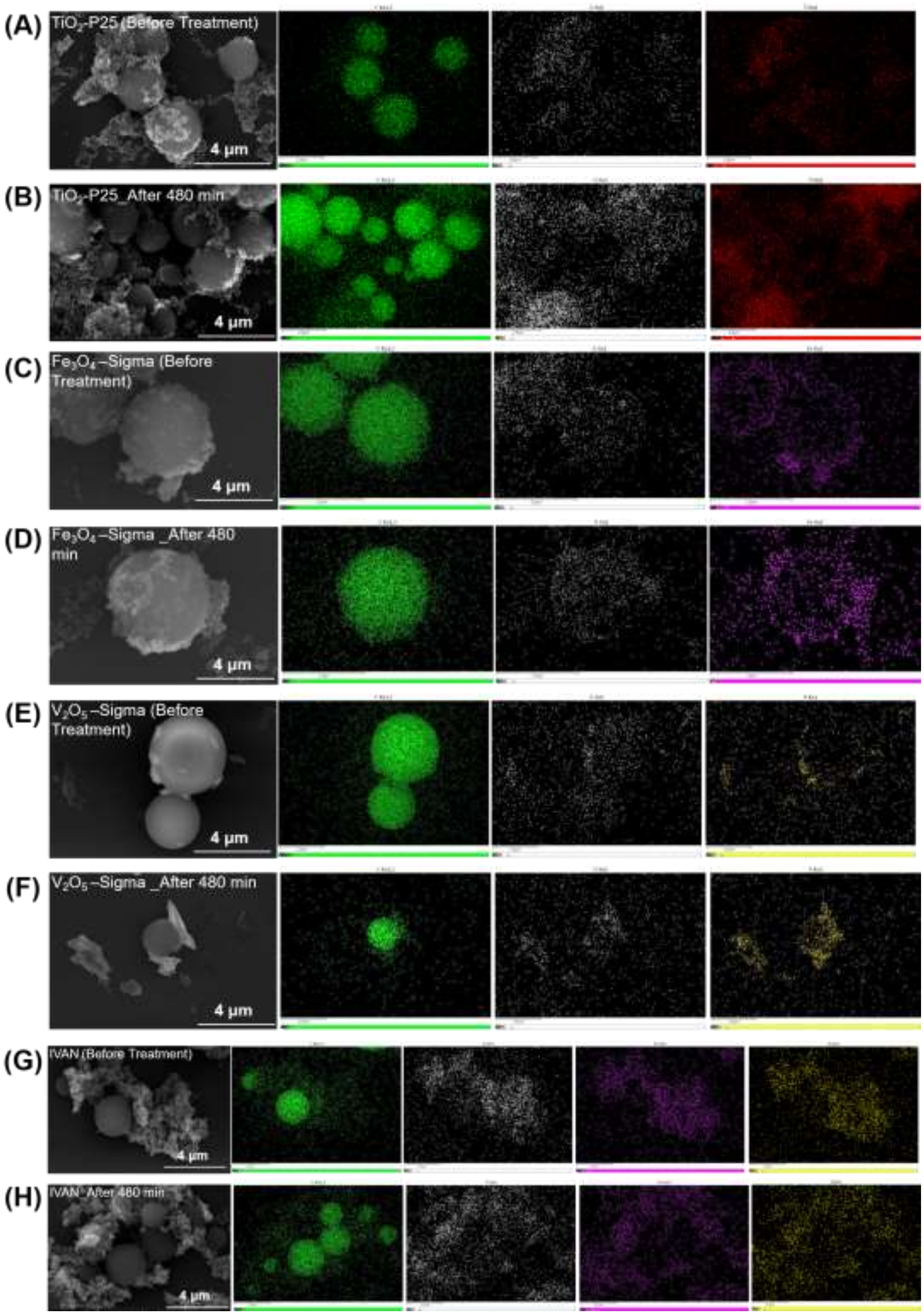

Figure S4: Representative HRSEM images with EDX mapping before (0 min) and after (480 min) the photocatalytic treatment for the degradation of PS MPs using the TiO_2-P25 (a &b), Fe_3O_4-Sigma (c & d), V_2O_5 Sigma (e & f), and IVAN (g & h). EDX mappings were colored

differently for each element, such as C (PS MPs) in green, Ti in red, Fe in violet, V in yellow, and O in white.

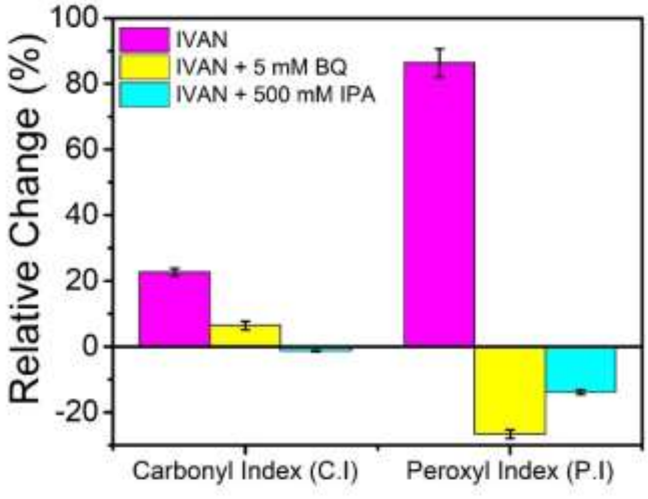

Figure S5: Relative changes in the CI and PI of IVAN with the addition of isopropyl alcohol (500 mM) and p-benzoquinone (5 mM) as a scavenger.

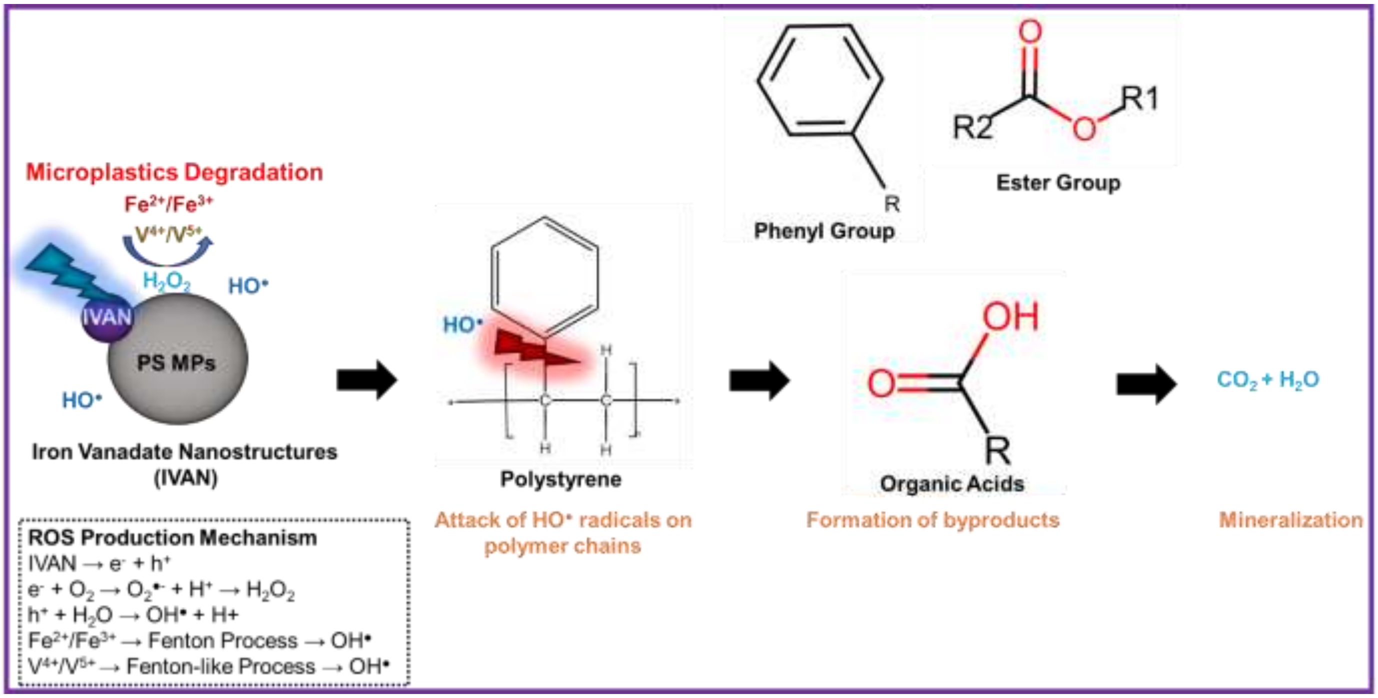

Figure S6: Mechanism of reactive oxygen species production and free radical attack site with chain breaking process.

(A)

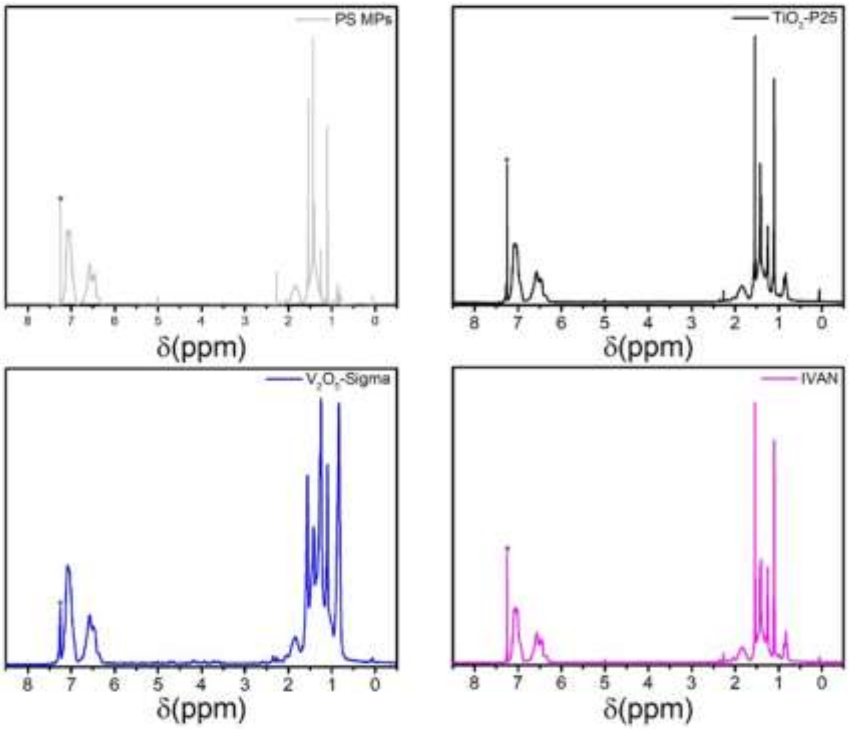
PS MPs
TiO2-P25
V2O5-Sigma
IVAN
δ(ppm)
δ(ppm)
δ(ppm)
δ(ppm)

(B)

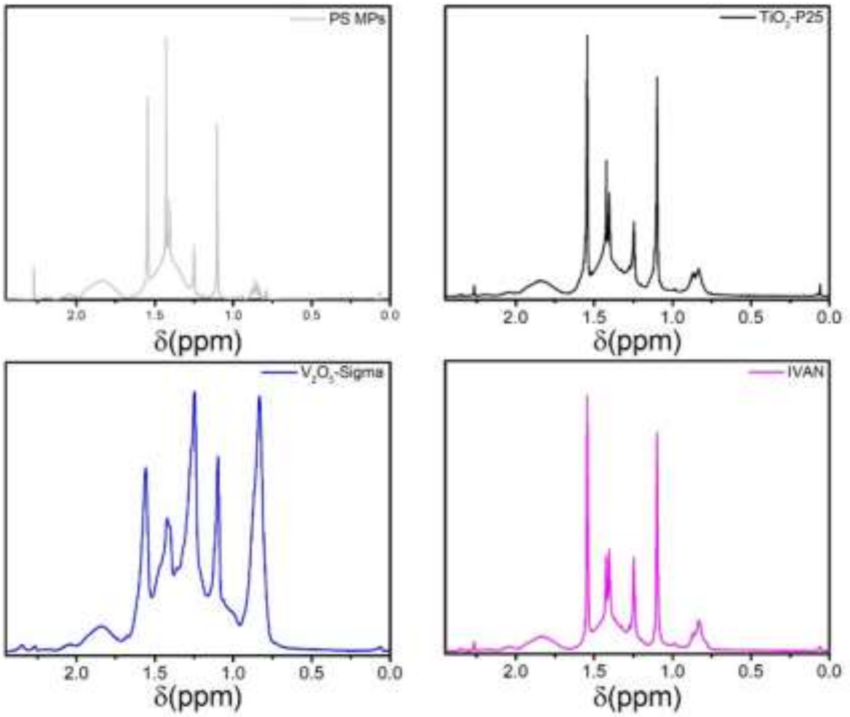
PS MPs
TiO2-P25
V2O5-Sigma
IVAN
δ(ppm)
δ(ppm)
δ(ppm)
δ(ppm)

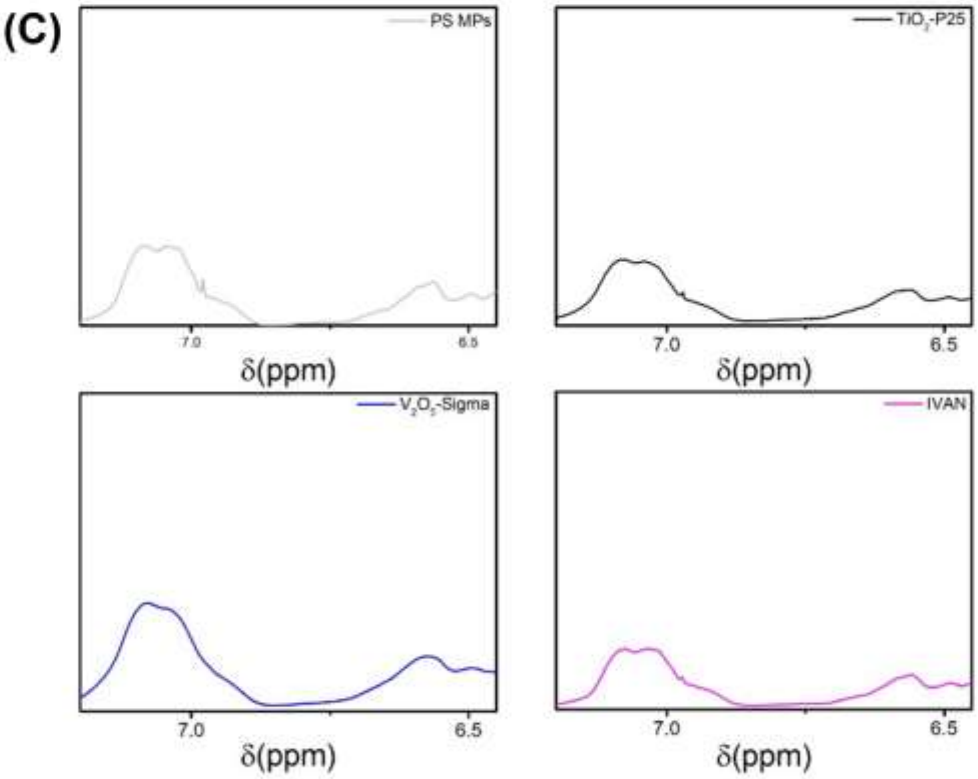

Figure S7: (A) ^{1}H NMR spectra of PS MPs, TiO_2-P25, V_2O_5-Sigma, and IVAN. (B) and (C) shows the zoomed NMR spectra between 2.45-0 ppm and 7.2-6.45 ppm, respectively. Solvent peaks are marked with an asterisk (*).

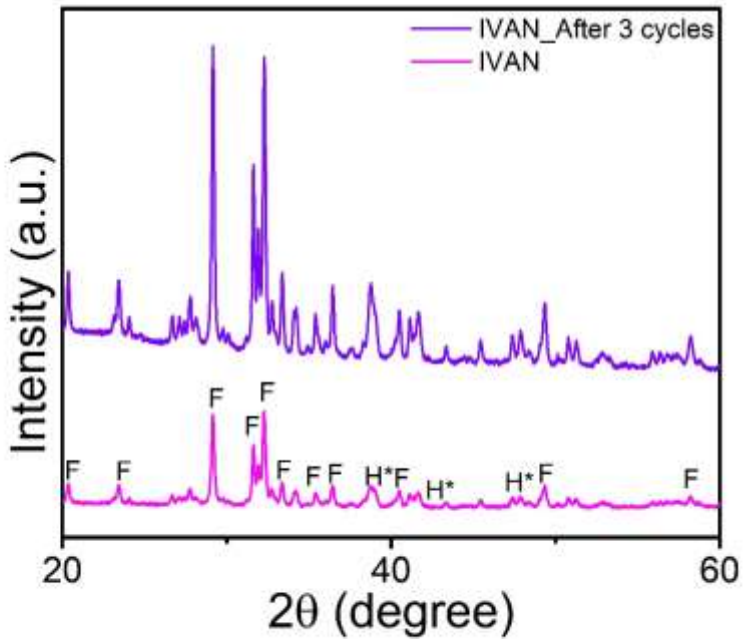

Figure S8: XRD patterns of IVAN after 3 photocatalytic cycles.

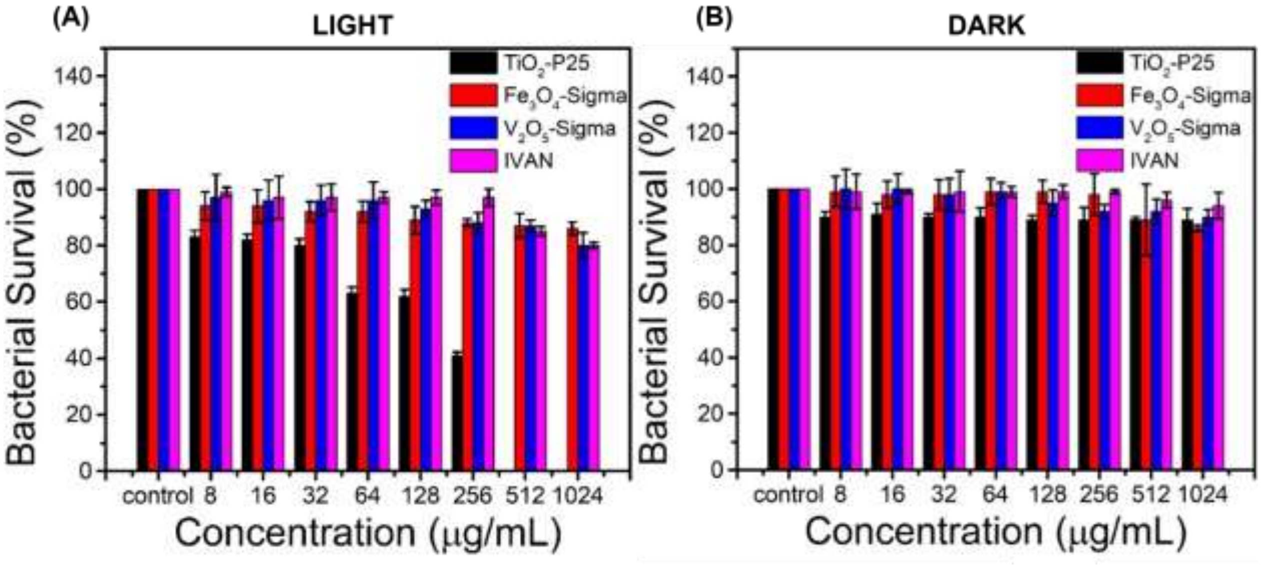

Figure S9: Antibacterial activity of the Fe_3O_4-Sigma, IVAN, TiO_2-P25, and V_2O_5-Sigma against planktonic forms of *S. aureus*. Tests were performed (A) after irradiation with the xenon lamp and (B) without irradiation (dark conditions).
